# Transcriptomic and evolutionary analysis of the mechanisms by which *P. argentatum*, a rubber producing perennial, responds to drought

**DOI:** 10.1186/s12870-019-2106-2

**Published:** 2019-11-13

**Authors:** Andrew D. L. Nelson, Grisel Ponciano, Colleen McMahan, Daniel C. Ilut, N. Ace Pugh, Diaa Eldin Elshikha, Douglas J. Hunsaker, Duke Pauli

**Affiliations:** 1 School of Plant Sciences, University of Arizona, Tucson, AZ 85721-0036 USA; 2 Western Regional Research Center, Agricultural Research Service, United States Department of Agriculture, Albany, California, 94710 USA; 3 Plant Breeding and Genetics Section, School of Integrative Plant Science, Cornell University, Ithaca, NY 14853 USA; 4 Biosystems Engineering, University of Arizona, Tucson, AZ 85721 USA; 5 Agricultural Engineering Department, Faculty of Agriculture, Mansoura University, Mansoura, Egypt; 6 USDA-ARS, Arid Land Agricultural Research Center, Maricopa, AZ 85138 USA

**Keywords:** Guayule, Transcriptome, Drought stress, Rubber biosynthesis, lncRNAs, Comparative genomic

## Abstract

**Background:**

Guayule (*Parthenium argentatum Gray*) is a drought tolerant, rubber producing perennial shrub native to northern Mexico and the US Southwest. *Hevea brasiliensis*, currently the world’s only source of natural rubber, is grown as a monoculture, leaving it vulnerable to both biotic and abiotic stressors. Isolation of rubber from guayule occurs by mechanical harvesting of the entire plant. It has been reported that environmental conditions leading up to harvest have a profound impact on rubber yield. The link between rubber biosynthesis and drought, a common environmental condition in guayule’s native habitat, is currently unclear.

**Results:**

We took a transcriptomic and comparative genomic approach to determine how drought impacts rubber biosynthesis in guayule. We compared transcriptional profiles of stem tissue, the location of guayule rubber biosynthesis, collected from field-grown plants subjected to water-deficit (drought) and well-watered (control) conditions. Plants subjected to the imposed drought conditions displayed an increase in production of transcripts associated with defense responses and water homeostasis, and a decrease in transcripts associated with rubber biosynthesis. An evolutionary and comparative analysis of stress-response transcripts suggests that more anciently duplicated transcripts shared among the Asteraceae, rather than recently derived duplicates, are contributing to the drought response observed in guayule. In addition, we identified several deeply conserved long non-coding RNAs (lncRNAs) containing microRNA binding motifs. One lncRNA in particular, with origins at the base of Asteraceae, may be regulating the vegetative to reproductive transition observed in water-stressed guayule by acting as a miRNA sponge for miR166.

**Conclusions:**

These data represent the first genomic analyses of how guayule responds to drought like conditions in agricultural production settings. We identified an inverse relationship between stress-responsive transcripts and those associated with precursor pathways to rubber biosynthesis suggesting a physiological trade-off between maintaining homeostasis and plant productivity. We also identify a number of regulators of abiotic responses, including transcription factors and lncRNAs, that are strong candidates for future projects aimed at modulating rubber biosynthesis under water-limiting conditions common to guayules’ native production environment.

## Background

Natural rubber is a crucial material with a myriad of uses and applications, making it invaluable to a wide range of industries, and contributing to its economic footprint of ~ 12.7 billion USD (DESA/UNSD). Natural rubber production, which is predominantly sourced from the rubber tree (*Hevea brasiliensis*), is currently threatened posing socioeconomic risks to industries relying on it as raw material (1). Because the species is clonally propagated and is grown as a geographically concentrated monoculture, it is vulnerable to diseases such as South American leaf blight (*Microcyclus ulei*), a fungal pathogen endemic to *Hevea*’s center of origin in the Amazon (2). Due to these growing concerns for the future stability of *Hevea* populations, scientists have continually searched for alternative sources of natural rubber (3,4). One such species, guayule (*Parthenium argentatum* A. Gray), has already been shown to be an attractive source of natural rubber that may be able to help address projected future shortages (3–6).

Guayule grows throughout northern Mexico and much of the American southwest and thus is naturally adapted to arid environments (6, 7). Because of this, producers first considered guayule as an alternative source of natural rubber in the early 1900’s. Subsequent utilization of guayule as a rubber source has progressed through multiple “boom and bust” phases largely influenced by world markets and import costs surrounding rubber from *H. brasiliensis* (6). Due to over a century of sporadic but intense efforts to harness guayule’s rubber producing potential, it is now understood that the crop has practical advantages over *Hevea*; for example, as a hypoallergenic alternative for those that have adverse reactions to latex rubber (8).

Crop scientists are now aware of unique challenges that guayule poses, particularly from a plant breeding perspective. Indeed, genetic improvement of guayule is complicated because the species has two different modes of reproduction and is able to exist as either facultatively apomictic, polyploid individuals or as sporophytic, self-incompatible diploid individuals (9–12). Due to this inherent biological complexity, a modern breeding approach that uses molecular techniques in tandem with traditional phenotypic selection may be the most effective way to increase the rate of genetic gain in the crop, particularly under stress conditions (13).

While the biological purpose for rubber biosynthesis and accumulation in the plant is unclear, its production is believed to be linked to the way the plant responds to abiotic stressors such as drought and temperature (14–17). Abiotic stress has been shown to elicit a dramatic and highly tissue specific reprogramming of the transcriptional profile in many plant systems (18). For instance, reproductive tissue in maize exhibits down regulation of genes associated with cell division and DNA replication during drought exposure, consistent with the observed delay in ear growth (19). In sunflower (*Helianthus annuus*), a naturally drought tolerant relative of guayule, exposure to drought conditions resulted in elevated levels of genes associated with osmotic adjustment in leaf and vasculature tissue (20), as well as decreases in genes associated with oil metabolism in the seed (21). While guayule exhibits similar drought tolerant characteristics as sunflower, it is unclear if it responds to stress in a similar molecular manner.

One critical, but until recently, overlooked aspect of the stress response in plants lies in the non-coding RNAs (microRNAs and long non-coding RNAs) that help sense and regulate the response to stress (22–24). These non-coding RNAs act at the pre- and post-transcriptional level to modulate expression and activity of other genes necessary for the stress response (25). Due to their highly specific expression patterns and species specificity sequence conservation, these transcript classes are important targets for understanding the unique ways in which plants have evolved to respond to changes in their environment (26–28). As such, all aspects of the transcriptome must be examined to fully understand the link between rubber biosynthesis and drought stress in guayule.

To uncover the molecular mechanisms that facilitate the drought response in guayule, we used a transcriptomic approach to identify differentially expressed transcripts between plants grown under both well-watered and water limited conditions. We used a phylogenetic approach to gain some insight into whether recent or more ancient gene duplications were contributing to the observed stress response. Finally, we uncovered a number of stress-responsive, long non-coding RNAs, several of which harbor conserved miRNA binding motifs, including two miRNAs with known roles in flowering and drought responses. These lncRNAs add an additional layer of regulatory complexity to the guayule drought response. Thus, we present a first glimpse at how guayule responds to drought and offer some molecular targets for plant breeders wishing to study the trade-off between rubber biosynthesis and water conservation.

## Results

### Examining the impact of drought at the transcriptome-wide level in guayule

**Fig. 1:**
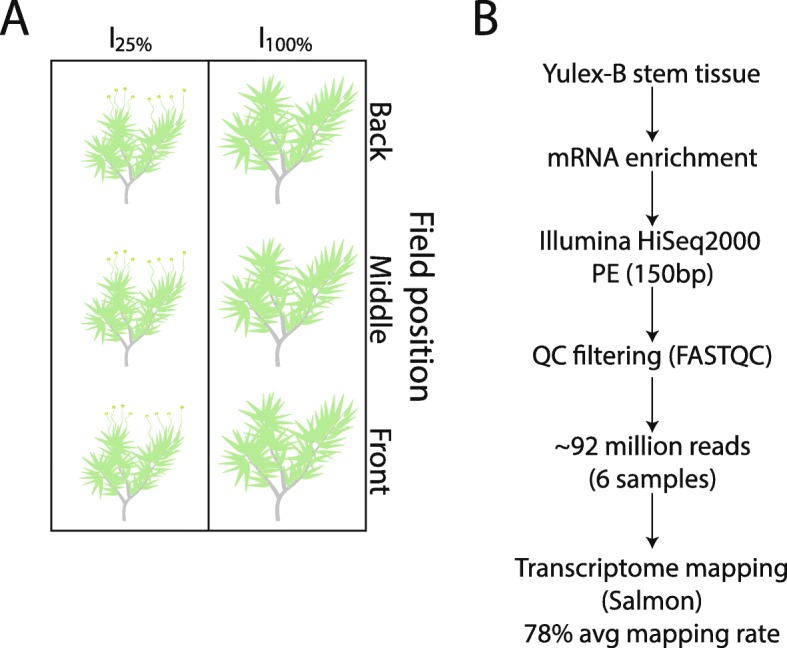
Irrigation and collection scheme for water-limited guayule. (**a**) Schematic representation of irrigation and collection conditions of field grown guayule. Note that guayule grown under water-deficit conditions (25% of control, with control receiving sufficient irrigation to meet measured evaporative soil water losses) were flowering whereas control plants were not. **(b)** Experimental design for transcriptomic profiling

Guayule is a drought tolerant species that has likely evolved a number of physiological mechanisms that enable it to mitigate the effects of drought prevalent in its native environment. To gain an understanding of what genes might be involved in guayule’s drought response mechanisms, we evaluated the guayule accession AZ-3 grown in plots for 29 months in Maricopa, Arizona having two contrasting irrigation regimes, I_100%_ and I_25%_ (Fig. 1a; (29, 30). The I_100%_ (or control treatment) was completely replenished with irrigation water, meeting measured evaporative soil water losses, while the I_25%_ received only 25% of the irrigation given to I_100%_. At the time of collection in March of 2015, the 29-month-old I_25%_ guayule plants were flowering in comparison to those grown at I_100%,_ which were not (Additional file 1: Figure S1). Stem tissue, the predominant location of guayule rubber biosynthesis, was collected from three biological replicates in each irrigation regime for transcriptomic analysis (Fig. 1b).

**Fig. 2:**
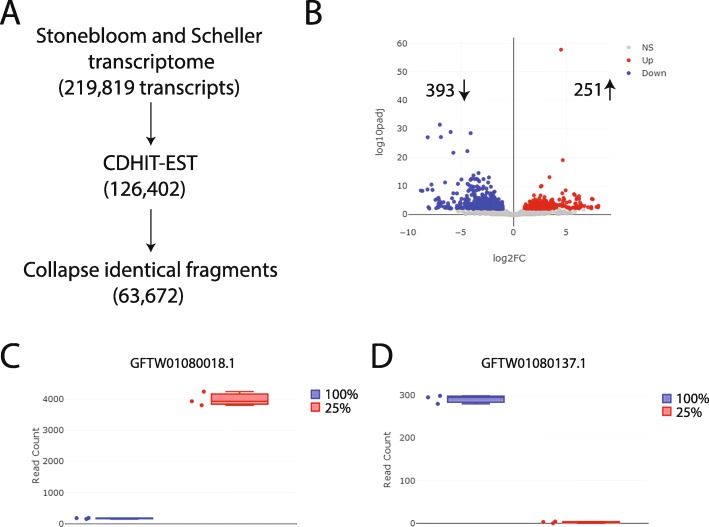
Transcriptomic comparison of plants grown under water-deficit conditions relative to control using a transcriptome-guided approach. (**a**) Schematic describing the approach taken to filter the Stonebloom and Scheller (2019) de novo assembled transcriptome. (**b**) Volcano plot representation of the transcripts differentially expressed under drought relative to control conditions. Log2 fold change (x-axis) is plotted relative to log10 adjusted *p*-value (y-axis). Transcripts upregulated under drought conditions and with an adjusted p-value < 0.01 are shown in red, whereas those downregulated are shown in blue. (**c**) Box and whiskers expression profile, as denoted by the number of reads mapped to the transcript (read count, y-axis), for the transcript most upregulated under water-deficit conditions (red bar, I_25%_). The three dots next to each bar represent the three biological replicates for each condition. (**d**) A similar expression profile for the transcript most down-regulated by water-deficit conditions

Given that no guayule genome is currently available for public use, we utilized a previously published de novo assembled transcriptome generated from a mixture of 150 and 300 bp reads (13) for read mapping. This transcriptome contains > 200,000 transcripts, suggesting the presence of incomplete or redundant (identical) transcripts. The presence of multiple fragments corresponding to the same transcript might confound our attempts to identify genes that are differentially expressed in response to limited water. The Stonebloom and Scheller transcriptome was filtered in two ways (Fig. 2a), collapsing the transcriptome from 219,819 transcripts to 63,672, a figure congruent with expectations. To ensure that filtering had not removed a significant number of actual transcripts, we mapped our RNA-sequencing data to both filtered and unfiltered transcriptomes and compared the number of reads that mapped to both. No differences were observed in mapping rates (~ 0.5% improvement in mapping to filtered set over unfiltered; Additional file 2: Table S1), suggesting that the filtered transcriptome would be sufficient for differential expression (DE) analyses.

Differentially expressed genes were identified by comparing the I_25%_ irrigation treatment to the I_100%_. Of the 63,672 transcripts, 42,711 were expressed (minimum of 0.5 TPM in all replicates) in the control conditions and 43,002 in the samples grown under the limited water. Of these, 251 transcripts were upregulated under the water-limited irrigation regime whereas 393 were downregulated (Fig. 2b and Additional file 3: Table S2; adjusted *p*-value of 0.01). The transcript most significantly upregulated in the water-limited treatment, GFTW01080018.1 (Fig. 2c), was expressed 23-fold compared to the control treatment (~ 9 –fold increase observed with qRT-PCR, Additional file 4: Figure S2). In contrast, the transcript most significantly downregulated, GFTW01080137.1 (Fig. 2d), was reduced more than 200-fold to near imperceptible detection levels, a value confirmed by qRT-PCR (Additional file 4: Figure S2).

**Fig. 3:**
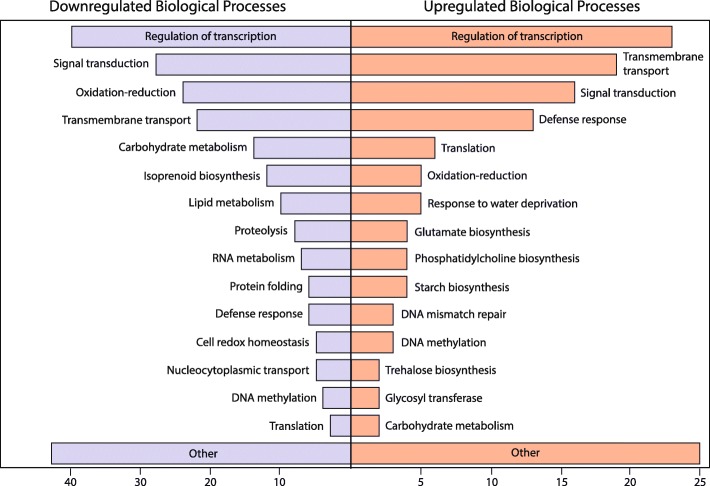
Functional analysis of differentially expressed transcripts. Biological processes inferred from gene ontological (GO) terms associated with either InterPro IDs or Arabidopsis orthologs were grouped into major categories. Note difference in scale of x-axis between down and up-regulated GO-terms

To gain an understanding of the cellular mechanisms that are involved in guayule’s response to drought, we performed a GO analysis of the significantly up- and down-regulated transcripts. An InterPro ID or shared similarity with an Arabidopsis protein-coding gene allowed us to infer biological processes for 273 of the 393 downregulated, and 163 of the 251 upregulated transcripts (Additional file 4: Table S3). Transcription factors (regulation of transcription) were the most abundant class of both up and down-regulated transcripts (Fig. 3). In agreement with previous data from drought-stressed plants, defense response, trehalose biosynthesis (31), glycosyltransferase activity (32, 33), and response to water deficit were among the processes more likely to be upregulated under the water-limited irrigation treatment, whereas isoprenoid/terpenoid biosynthesis, carbohydrate metabolism, and lipid metabolism processes were more likely to be downregulated (Fig. 3).

Next, the most differentially expressed transcripts were assessed. The most significant, highly upregulated transcript, GFTW01080018.1, appears to be orthologous to the Arabidopsis PIP2s (specifically PIP2A, B, and C; Additional file 6: Figure S3), a family of aquaporins important for hydraulic regulation (34). Despite the recovery of numerous PIP2 paralogs in the genomes of *Helianthus annuus* and *Lactuca sativa*, two close relatives of guayule within the Asteraceae (35); Additional file 6: Figure S3), and three paralogs in the guayule transcriptome, only one aquaporin was differentially expressed in response to water deficit (I_25%_). The most significantly down-regulated transcript, GFTW01080137.1, shares sequence similarity to Arabidopsis Cold Regulated Gene 27 (COR27; AT5G42900). Interestingly, in Arabidopsis, COR27 and another cold regulated gene with little sequence similarity, COR28, are positive regulators of flowering (36). In guayule, putative orthologs for both COR27 and COR28 (GFTW01080137.1 and GFTW01127972.1, respectively) are both significantly repressed under water limited conditions, despite the near uniform flowering that was observed for these plants (Additional file 1: Figure S1). Finally, GFTW01028919.1, the transcript that displayed the greatest decrease in transcription (although not the most significant), at > 900-fold (adjusted *p*-value <2E-12; Additional file 7: Figure S4) is a putative ortholog of Arabidopsis Terpene Synthase 3 (AT4G16740) and is one of 12 downregulated guayule transcripts involved in isoprenoid/terpenoid biosynthesis (Fig. 3). In sum, guayule’s transcriptomic response to water-limited conditions includes a dramatic increase in aquaporin production and defense response genes, as well as a decrease in terpenoid biosynthesis, carbohydrate metabolism, and oxidation reduction mechanisms.

### Examining the evolutionary history of duplicated drought-responsive transcripts

The GO-term analysis revealed that some of the differentially expressed guayule transcripts displayed similarity to the same Arabidopsis gene, suggesting one of three possibilities: 1) an ancient expansion in a stress-responsive gene family, 2) that the transcripts are paralogs that emerged following the cross-hybridization and polyploidy event that gave rise to AZ-3, or 3) that the transcripts contain the same functional domain but bear no phylogenetic relationship. Specifically, 127 guayule stress-responsive transcripts clustered, in sets of 2–4 transcripts each, with 56 Arabidopsis genes. For example, the downregulated guayule terpene synthase ortholog (GFTW01028919.1) groups with AT4G16740 along with two other guayule transcripts (GFTW01072004.1 and GFTW01017460.1). We first determined if the guayule transcripts were indeed the product of a gene duplication by examining codon-guided multiple sequence alignments. Transcripts associated with roughly half (*n* = 27) of the Arabidopsis gene clusters either did not share a recent evolutionary past (sequence identity < 50%) or there was not enough evidence to support a gene duplication (e.g., guayule gene fragments that did not overlap one another in the alignment). The three guayule transcripts within the terpene synthase cluster with AT4G16740 shared sufficient sequence similarity to proceed forward to phylogenetic analysis, whereas three guayule transcripts that shared similarity with an Arabidopsis mitogen-activated protein kinase (MAPK16, AT5G19010) exhibited little to no similarity outside of the kinase domain and were not considered further.

**Fig. 4:**
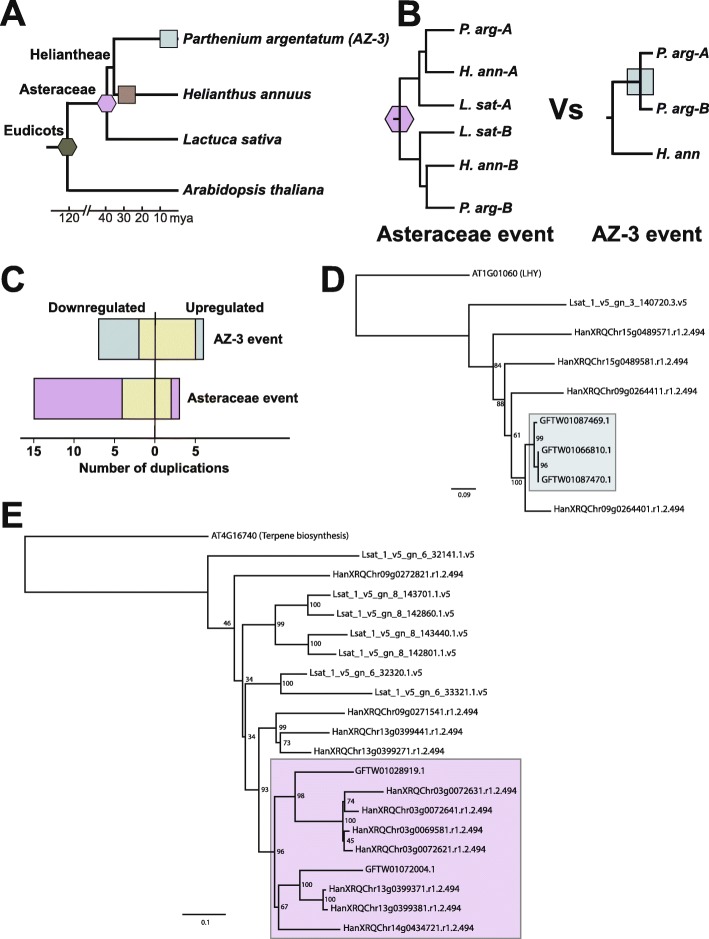
Phylogenetic inference of the timing of duplication for differentially expressed guayule transcripts. (**a**) Chronogram of the four species used to build gene trees for this analysis. Placement of markers representing whole genome triplication (hexagons) and duplication (squares) indicates whether an event occurred in a common ancestor and is therefore shared (e.g. purple hexagon indicates a genome triplication event in the last common ancestor to all Asteraceae), or if it is species-specific (e.g., the light blue square for guayule represents the duplication event in the accession examined in this study, AZ-3). (**b**) The two phylogenetic models used to infer timing of the duplicated transcripts observed in guayule. Left, duplicated guayule transcripts, represented by *P. arg-A* and *-B* are sister to *H. annuus* paralogs, represented by *H. ann-A* and *H. ann-B* and thus likely originated from the whole genome triplication event at the base of the Asteraceae (purple hexagon). Right, guayule paralogs are sister to one another in the gene tree and then to a *H. annuus* ortholog, suggesting a guayule specific duplication event (light blue square). (**c**) Bar plot indicating the number of differentially expressed guayule transcripts associated with each duplication event. Duplication events inferred to have arisen specifically in guayule (AZ-3) are shown in light blue, whereas those likely originating from the ancient Asteraceae hexaploidy event are shown in purple, using the same color scheme from a and b. Pseudogenization of one of the guayule paralogs is indicated by the tan bar. (**d**) Gene tree representing an AZ-3 specific duplication event (blue box). (**e**) Gene tree representing an Asteraceae event (purple box). In d and e, gene trees were rooted using the Arabidopsis ortholog

To determine the timing of the guayule gene duplication events associated with the remaining 29 Arabidopsis gene clusters, we took a comparative and evolutionary approach, searching the genomes of sunflower (*H. annuus*; (35)) and lettuce (*L. sativa*; (37)) for homologs to the stress-responsive guayule transcripts and their putative Arabidopsis orthologs. We then inferred phylogenies for each of these gene families to determine when the observed gene duplication occurred. Two whole genome triplication events are shared between sunflower and guayule, with an additional, species-specific whole genome duplication event occurring in each species (Fig. 4a). Thus, we examined the resulting phylogenies for two patterns that would indicate that the guayule transcripts were the result of an Asteraceae (or earlier) duplication event (Fig. 4b, left; “Asteraceae event”). In this scenario, each of the guayule transcripts would be immediately-sister to a sunflower gene. In the event that the transcript duplication was AZ-3 specific, we would observe the duplicated transcripts first sister to each other and then to a sunflower gene (Fig. 4b, right; “AZ-3 event”). Of the 20 Arabidopsis gene clusters comprised of down-regulated guayule transcripts, 13 contained transcripts where the gene duplication was inferred to be an Asteraceae event (Fig. 4c, purple bar), 7 arose from an AZ-3 event (Fig. 4c, blue bar), and two gene clusters contained both types of duplication events. Of the nine Arabidopsis gene clusters comprised of up-regulated guayule transcripts, three of the paralogs arose from an Asteraceae event, whereas six where AZ-3 specific (Fig. 4c). One example of a AZ-3 event can be seen in the putative guayule orthologs of AT1G01060 (LHY), a transcription factor that regulates flowering and circadian rhythm (Fig. 4d, blue box). These transcripts, all of which were significantly upregulated, fall sister to one another in the phylogeny with strong bootstrap support. In contrast, the terpene synthase gene cluster contained two guayule transcripts that were each sister to multiple sunflower genes (Fig. 4e, purple box).

Duplication and expression do not necessarily imply that the resulting transcript is capable of encoding for a protein. Pseudogenization or neo-functionalization of a locus (protein-coding gene - > long non-coding RNA) can occur through the disruption of a protein-coding gene’s open reading frame (ORF). We examined each of the gene clusters for loss of ORF integrity in at least one (but not all) of the duplicate guayule transcripts. We found that 6/20 of the down-regulated gene clusters had experienced a pseudogenization event that left them with a single protein-coding gene, whereas 7/9 up-regulated gene clusters were left with a single protein-coding transcript (Fig. 4c, tan bars). Thus, it appears that a number of stress-responsive paralogs with intact ORFs have been retained through multiple speciation events, suggesting they may help guayule mount a response to drought conditions.

### A role for long non-coding RNAs in guayule’s drought response

**Fig. 5:**
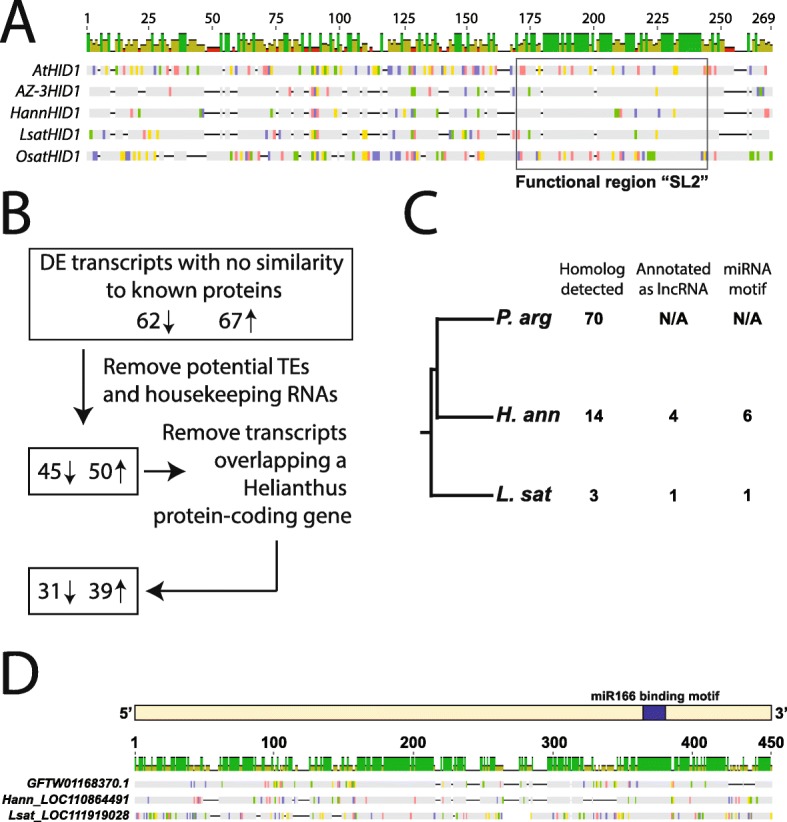
Identification and function inference of guayule stress-responsive lncRNAs. (**a**) Graphical representation of a multiple sequence alignment (MSA) of guayule HID1, along with sequence homologs from Arabidopsis (AtHID1), sunflower (HannHID1), lettuce (LsatHID1), and rice (OsatHID1). 100% sequence identity between all sequences in the MSA are represented by green in the coverage bar across the top. (**b**) Experimental design to identify putative guayule lncRNAs. “Known proteins” refers to proteins with annotated domains or that are found in the InterPro database. TEs = transposable elements. (**c**) Phylogenetic representation of the number of sequence homologs identified for the guayule lncRNAs. Number of lncRNA sequence homologs annotated as a lncRNA in either *H. annuus* or *L. sativa* is shown. Number of conserved guayule lncRNAs for which a miRNA binding motif is conserved is also indicated. (**d**) Graphical representation of an MSA of the putative miRNA sponge, GFTW01168370.1, with the 100% conserved miRNA binding site shown by the blue box along the top of the alignment. The corresponding lncRNA IDs for sunflower and lettuce are shown in this alignment

The identification of stress-responsive transcripts that are no longer protein-coding raises the possibility of uncovering long non-coding RNAs (lncRNAs) that are also differentially expressed under the water-limited irrigation regime. While not as extensively studied in plants as in vertebrate systems, a number of plant lncRNAs have been reported to differentially expressed in response to abiotic and biotic stress (38–42), where, among many functions, they can act as regulators of transcription, microRNA sponges, and influence alternative splicing (25,43,44). Although not differentially expressed under the imposed irrigation treatments, a homolog of the deeply conserved light responsive lncRNA, *HID1* *(*45*)*, was present in the guayule transcriptome (Fig. 5a). As expected based on prior analyses, the protein interaction domain annotated as SL2 was highly conserved between Asteraceae, Arabidopsis, and rice (Fig. 5a), suggesting a potentially shared role for this lncRNA across flowering plants. In addition, the identification of a guayule *HID1* demonstrates that the Stonebloom and Scheller transcriptome captured polyadenylated lncRNAs as well as protein-coding transcripts.

To identify putative lncRNAs, we focused on the set of differentially expressed transcripts that bore no similarity to any known protein domains (Fig. 5b). We then removed potential transposable elements (TEs) and known housekeeping RNAs (rRNAs and spliceosomal RNAs). To be conservative in our lncRNA identification, we also removed any transcripts that overlapped a protein-coding gene in the *H. annuus* genome, as these guayule transcripts may reflect incompletely assembled protein-coding genes resulting from technical difficulties of de novo transcriptome assembly. Following these filters, we recovered 31 putative lncRNAs that were down-regulated and 39 that were up-regulated in response to drought (a complete list can be found in Additional file 8: Table S4).

We then took an evolutionary approach to identify putative lncRNAs for which we could recover sequence homologs in other species under the premise that conservation implies functionality (26). Of the 70 guayule putative lncRNAs, we identified a sequence homolog for 14 in the sunflower genome (Fig. 5c). We uncovered evidence of conservation for three lncRNAs in the lettuce genome, suggesting that these loci emerged at least ~ 39 million years ago. Four of the fourteen sunflower conserved lncRNAs were also annotated as lncRNAs in that system, with one also annotated as a lncRNA in lettuce, lending additional confidence in their lncRNA designation (Fig. 5c).

Next, an attempt to assign a function to these putative lncRNAs beyond “stress-responsive” was made. Our experimental design lacked depth to attempt a “guilt-by-association” analysis, and the absence of a guayule genome precludes the association between a lncRNA and the neighboring protein-coding gene it might regulate. Therefore, we focused on whether the set of guayule lncRNAs might be involved in sequestering miRNAs away from their intended targets, or in miRNA or phasiRNA, biogenesis. Using psRNAtarget (46), we predicted whether miRNAs might bind to the 14 lncRNAs for which we identified sequence homologs in sunflower. We then scanned the homologous locus in sunflower (and in lettuce) for conservation of the miRNA binding site. Using this approach we identified six lncRNAs with conserved miRNA binding sites (Fig. 5c; Additional file 8: Table S4). One of the guayule lncRNAs conserved and annotated as a lncRNA in both sunflower and lettuce, GFTW01168370.1, harbors a completely conserved binding site for miR166 (Fig. 5d), a microRNA associated with tissue development and whose knockdown in Arabidopsis leads to an enhanced drought response (47). As a miRNA sponge, GFTW01168370.1 would act to recruit miR166 away from its intended target, in short mimicking the knockdown response reported in Arabidopsis. Thus, within the dataset of drought-responsive transcripts, a subset were identified that showed the hallmarks of being lncRNAs. Several of these lncRNAs contain conserved miRNA binding sites, with one in particular likely helping to mediate the guayule drought response.

## Discussion

### Transcriptome analyses reveal a suite of drought-responsive genes in guayule

As a perennial shrub native to the American Southwest and northern Mexico, guayule is well adapted to long periods of little to no water. Using next-generation sequencing, we examined the molecular mechanisms by which guayule responded to simulated drought conditions via imposed irrigation treatments. By examining stem tissue, the primary location of rubber biosynthesis in guayule, we were also able to consider the impact of drought on this metabolic pathway. We performed our analyses using a published transcriptome for guayule, taking steps to collapse potential isoforms and miss-assembled transcripts. As expected, we identified a number of differentially expressed transcripts involved in signal transduction pathways (e.g., protein phosphorylation), transcriptional regulation, and transmembrane transport. We identified more than 20 up or down-regulated transcripts with similarity to Arabidopsis transcription factors associated with circadian clock regulation. Interestingly, many of these transcripts are annotated as cell-to-cell mobile in Arabidopsis (48), perhaps indicating that our transcriptomic analysis in stem tissue is generating a snapshot of circadian regulation occurring elsewhere in the plant. Regardless, while drought conditions dramatically impact both flowering and the circadian clock in guayule, due to the abundance of transcripts, it is unclear which transcript might be the regulator/sensor that is connecting drought to flowering.

The most upregulated guayule transcript is orthologous to the Arabidopsis aquaporin PIP2 family. Interestingly, despite recent duplications in close relatives, sunflower and lettuce, that are likely shared with guayule, we only observed differential expression for a single aquaporin out of three observed in the transcriptome, suggesting that it is the key regulator of water transport in stem tissue. We also observed twelve transcripts associated with rubber biosynthesis that were down-regulated under water-limited conditions. Although guayule rubber biosynthesis is known to be induced by cold temperatures, little is known about the mechanistic impact drought has on this pathway. However, given the abundance of terpene biosynthesis-associated transcripts and their almost complete down-regulation suggests that guayule modulates precursors to the rubber biosynthesis pathway when faced with water deficit conditions. This is in agreement with the observation that I_100%_ plants contained twice the rubber content of those grown at I_25%_ even though water use efficiency was equivalent (29).

### WGD events have added to the complexity of the guayule drought response

Gene duplication, when the resulting duplicate is retained, can result in increased nuance in how plants perceive and respond to abiotic stress (49). The presence of duplicated transcripts in guayule are not surprising, given the multiple reported whole genome duplication (WGD) events leading up to the speciation event of guayule (35). A whole genome triplication event occurred at the base of the Asteraceae and is shared among all family members. More recently, a whole genome duplication has been observed in the formation of the guayule accession used in this analysis, AZ-3. AZ-3 is a complex polyploid formed by the likely hybridization of diploid *P. argentatum* and an unknown Parthenium species. Tetraploid guayule reportedly has increased biomass, rubber yield, and vigor compared to its diploid relatives. Thus, both of these polyploidization events raise the possibility that some of the duplicated genes may be mediating a successful response to drought stress or are contributing to increased vigor in the species.

We searched for evidence of duplication in the stress-responsive transcripts using a parsimony based approach to infer when those duplications occurred. It should be noted that we are not observing all duplicate genes here, only the ones that continue to be stress-responsive following duplication. These transcripts likely retain conservation in their regulatory domains (e.g., promoter elements), but in the absence of a genome, we focused on retention of protein-coding capacity. We were able to infer duplication events for 29 clusters of 68 stress-responsive guayule transcripts, with most (18/29) duplication events shared across Asteraceae. ORFs were retained in a majority of these transcripts (16/29), which, when combined with the shared pattern of differential expression between paralogs and their deep conservation, suggests that these duplicates are functional. However, as most of the observed retained duplicates appear to be shared across Asteraceae, they likely cannot explain the vigor associated with tetraploid guayule.

### LncRNAs are helping to mediate the drought response in guayule

Long non-coding RNAs add an additional layer of complexity to plant stress responses through their ability to act as pre- and post-transcriptional regulators of gene expression. Interestingly, we recovered a homolog of *HID1*, a lncRNA that helps mediate shade avoidance in Arabidopsis. Although HID1 is conserved across land plants, this is the first Asterid homolog identified. In agreement with previous reports on *HID1* conservation, guayule *HID1* was conserved in the 5′ region believed to be important for protein-binding. Given the role of *HID1* in light signaling it is perhaps not surprising that its expression was not responsive to drought. However, we were able to identify 70 putative lncRNAs that were differentially expressed in response to drought, 14 of which were conserved in the sunflower genome. De novo transcriptome assembly routinely produces fragmented transcripts with disrupted ORFs that would appear to look like a lncRNA. Thus, we took a more conservative approach than is typically taken when a reference genome is available by filtering out any transcripts that shared sequence similarity with protein-coding genes from related species. Four of the sunflower-conserved lncRNAs were also annotated as lncRNAs in sunflower, lending further support to their classification in guayule. Based on conservation and their stress-responsiveness, we would predict that these lncRNAs are likely functioning to modulate the drought response in guayule.

Functional prediction for lncRNAs is difficult in the absence of genomic context clues or without the ability to apply guilt-by-association strategies through many experimental time points or conditions. Thus, we focused on one functional class of lncRNA, that of miRNA sponge/precursor, as miRNA binding sites are fairly easy to predict computationally. Again, using sequence conservation as a means of boosting predictive confidence, we identified conserved miRNA binding sites in six guayule lncRNAs. One of these putative miRNA sponges in particular harbors a binding site for miR166, a microRNA involved in vegetative growth, floral morphogenesis, and regulating responses to salinity and drought. The lncRNA containing the miR166 binding site is upregulated under drought conditions and therefore could be mediating either the observed floral transition or the drought response.

## Conclusions

As a drought tolerant, rubber producing perennial crop, guayule represents a remarkable natural resource for meeting industrial demands for raw products. In the present work, a transcriptomic and comparative evolutionary analysis approach was taken to identify and characterize the molecular response of guayule to drought-like conditions. We found that rubber biosynthesis-associated transcripts were dramatically down-regulated in the plants subjected to water-limited conditions in comparison to the plants in the well-watered control treatment. These results demonstrate that even given guayule’s inherent drought tolerance, there is a molecular trade-off occurring between rubber biosynthesis and the plants ability to maintain hydration status and homeostasis. These findings suggest that water and other crop inputs need to be optimized with respect to rubber yield to find an economic balance for potential producers.

## Methods

### Plant growth and tissue collection

Guayule (*P. argentatum* AZ-3) seed was obtained from the USDA-ARS National Plant Germplasm System (NPGS; https://www.ars-grin.gov/) using ID PI 599676. Guayule plants were grown in the field under subsurface drip irrigation at the University of Arizona, Maricopa Agricultural Center in Maricopa, Arizona as described in (29). On the day of final harvest when plants were 29 months-old (March 2015), 10–15 mm diameter-stem segments from each plant were harvested and immediately frozen in liquid nitrogen and then stored at − 80 °C until used. Three biological replicates for each treatment were harvested.

### RNA extraction and Illumina library preparation

Approximately 2 g of stem tissue was used for total RNA extracted following the Laudencia et al. 2007 (50) protocol with the following modifications: (i) acid phenol:chloroform MB grade (Ambion, USA) was used for the phenol:chloroform extraction step; (ii) the precipitated RNA was further cleaned with Qiagen RNeasy Plant Mini Kit (Qiagen, USA); and (iii) the cleaned RNA was treated with DNA-*free*™ kit (Ambion, USA). PolyA-RNA was prepared employing Qiagen RNeasy/QIAshredder protocols (Qiagen, USA). RNA-sequencing libraries were prepared using the KAPA stranded mRNA-seq kit for Illumina (KK8420) according to the manufacturer’s protocol (KR0960 - v3.15). RNA-sequencing was performed on the Illumina HiSeq2000 with 150 bp paired-end reads. A total of 98,430,986 reads was generated for the six samples.

### Transcriptomic analysis

A condensed version of the Stonebloom and Scheller transcriptome was generated by initially filtered using CD-HIT-EST v.4.6.8 (51) with a global sequence identity of 1 (100%). To identify potentially identical transcripts that contained a single mis-aligned read, 150 nts were removed from either the 5′ or 3′ end of the transcript, and if the resulting transcript was greater than 150 nts, was used as query in a BLASTn (52) against all other transcripts. Hits against self were removed, and then all other hits with 100% coverage of one of the sequences, as well as 100% identity, were collapsed into one transcript, with the longest transcript being retained. Read mapping and quantification was performed using Salmon v0.81 (53) in CyVerse’s Discovery Environment (54). Quantified reads were prepared for differential expression analysis using the tximport (55) package in R. Differential expression was determined using DESeq2 (56) with an adjusted *p*-value of 0.01 as the cutoff for significance.

### Quantitative PCR (qPCR) analysis

Stem bark tissue was the source of RNA for qPCR analysis. For each of three biological replicates (of both water-deficit and well-watered plants), total RNA was extracted with RNAqueous™ kit (Invitrogen, USA) and traces of DNA removed with DNA-free™ kit (Life Technologies, USA). Two micrograms of total RNA were the template for oligo (dT)_20_-generated cDNA with SuperScriptIII First-Strand Synthesis System for qPCR (Life Technologies, USA) following manufacturer instructions. The qPCR reactions were carried out using Applied Biosystems 7500 Fast Real Time PCR System and SYBR Green chemistry (Life Technologies, USA) in 20 μl volume reactions containing 400 ng of template cDNA, 900 nM of each forward and reverse primer, 10 μl of Fast SYBR® Green Master Mix, and water as needed. The following combinations of forward/reverse primers were used: for GFTW01080018.1, 5′- TGCCGTATTCATGGTTCACTTG -3′ / 5′- GGGCCGGGTTGATTCC − 3′; for GFTW01080137.1, 5′- TTTGTGGAGCAGGAGGAGAG-3′ / 5′-GCCAGATGAAACTGTATCAGAGC-3′; for GFTW01028919.1, 5′-ACTTCAAAGGTCGTTCCAAGAC-3′ / 5′-TGCCTCGCATTTTTCTCCAG-3′; and for *Pa18S*, 5′- TACTATGGTGGTGACGGGTG -3′/5′- ATTGTCACTACCTCCCCGTG − 3′. Thermocycler temperature regime was 95 °C for 20s, followed by 40 cycles of 95 °C for 3 s and 60 °C for 30s. Data were analyzed using the 7500 Fast System Detection Software (Life Technologies, USA) with manually set threshold. Expression of each target gene was calculated with the Livak and Schmittgen 2001 method (57), normalized to expression of the endogenous reference gene *Pa18S*, and then to its expression in a calibrator (well-watered control plant). Four technical replicates qPCR reactions were run for each target gene, and the whole experiment was performed twice using the same RNA but freshly synthesized cDNA.

### Functional analysis

GO terms for the differentially expressed transcripts were identified using BLAST2GO (58). First, BLASTx was performed against a database of Arabidopsis protein-coding genes with an e-value of 1E-3 and word size of 3. Protein domains were identified using InterProScan with default parameters. For guayule transcripts sharing similarity with an Arabidopsis protein-coding gene as determined by BLASTx analysis, but for which no functional annotation was obtained through BLAST2GO, we extracted Biological Processes directly from TAIR (59).

### Duplication event timing and phylogenetic analysis

To determine timing of duplication, gene families were first generated by identifying sequences in the *H. annuus* (CoGe ID 37147) and *L. sativa* (CoGe ID 37106) genomes that shared sequence similarity with both the Arabidopsis and guayule sequences using CoGe BLAST with default parameters and an E-value of 1E-10 (60). Coding sequences were extracted from the top five unique loci in each genome using CoGeBLAST’s view FASTA feature. Sequences were aligned using MAFFT (61) in Geneious (62). The 5′ and 3′ UTRs of guayule transcripts were trimmed based on the multiple sequence alignment so that all sequences started with an “ATG” and ended with a stop codon. These alignments were then used to infer phylogenetic relationships with RAxML (v7.2.8, (63)) with the GTR GAMMA substitution model and 100 bootstraps. Trees with poor support (< 70) specifically at the guayule-sunflower node were realigned with fewer sequences or different MAFFT parameters until the support increased above 70. The sister branch to the query guayule sequence, whether it was sunflower or a guayule paralog, was used to infer timing of the duplication event based on the known organismal phylogeny.

### LncRNA identification, conservation, and functional assessment

Differentially expressed lncRNAs were identified by first filtering out differentially expressed transcripts that displayed any similarity with known proteins or annotated protein domains (BLASTx, 1E-3). Transcripts were then filtered using Evolinc (64), filtering based on length, coding capacity (using CPC; (65)), and similarity to annotated proteins from the same species (using the set of differentially expressed transcripts predicted to be protein-coding). Sequence homologs for these lncRNAs were identified in the sunflower and lettuce genomes using CoGe BLAST, with an E-value of 1E-20 (26). Guayule transcripts for which a sequence homolog in another species corresponded to an annotated protein-coding gene were removed. These cleared transcripts were then scanned for miRNA motifs using psRNATarget (2017 update). Putative miRNA motifs were examined for conservation using multiple sequence alignments generated by MAFFT and visualized in Geneious.

## Supplementary information

**Additional file 1: Figure S1.** Representative image of water-restricted guayule flowering relative to non-flowering control plants of the same age.

**Additional file 2: Table S1.** Mapping rate comparison between filtered and unfiltered guayule transcriptome.

**Additional file 3: Table S2.** Complete results of differential expression analysis.

**Additional file 4: Figure S2.** qRT-PCR validation of the most differentially expressed guayule drought responsive genes.

**Additional file 5: Table S3.** Complete list of GO results for differentially expressed transcripts.

**Additional file 6: Figure S3.** Gene tree of the guayule aquaporin PIP2A ortholog.

**Additional file 7: Figure S4.** Gene tree of the guayule gene GFTW010289191.1, a putative ortholog of Arabidopsis AT4G16740, a gene involved in terpene biosynthesis.

**Additional file 8: Table S4.** List of putative guayule lncRNAs organized by conservation within the Asteraceae and presence of miRNA binding motifs.

## Data Availability

RNA-seq data have been uploaded to NCBI’s SRA under the BioProject ID PRJNA400611.

## Abbreviations

bp: base pairs
DE: differential expression
GO: gene ontology
HID1: Hidden Treasure 1
LHY: Late elongated hypocotyl
lncRNA: long non-coding RNA
miRNA: microRNA
mRNA: messenger RNA
nt: nucleotide
phasiRNA: phased, secondary, small interfering RNAs.
PIP: Plasma membrane intrinsic protein
rRNA: ribosomal RNA
TPM: transcript per kilobase million
USD: United States Dollars
