# Supplementary figures and images for: Transcriptomic and evolutionary analysis of the mechanisms by which P. argentatum, a rubber producing perennial, responds to drought
Source: BMC Plant Biol. 2019 Nov 13;19:494. doi: 10.1186/s12870-019-2106-2 (PMC6854645; doi:10.1186/s12870-019-2106-2)

$I_{25\%}$

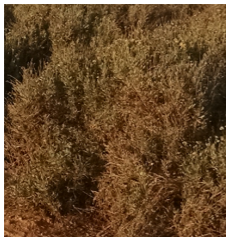

$I_{100\%}$

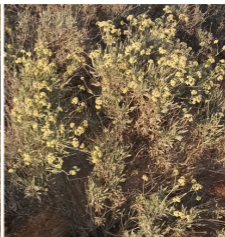

Additional file 1: Figure S1

Supplement: Supplementary file 1 — Additional file 1: Figure S1. Representative image of water-restricted guayule flowering relative to non-flowering control plants of the same age. [file 12870_2019_2106_MOESM1_ESM.pdf]

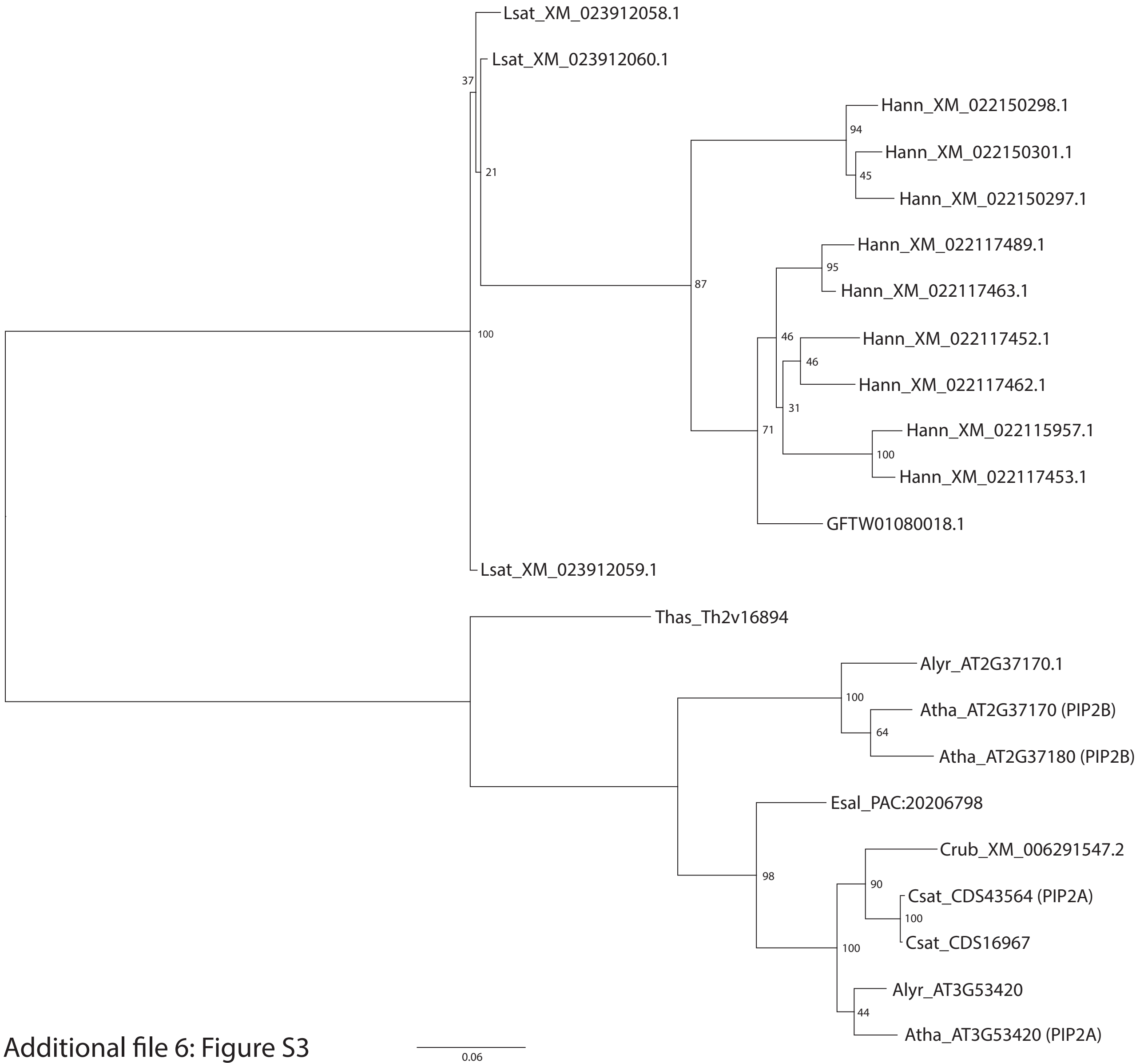

Supplement: Supplementary file 6 — Additional file 6: Figure S3. Gene tree of the guayule aquaporin PIP2A ortholog. [file 12870_2019_2106_MOESM6_ESM.pdf]

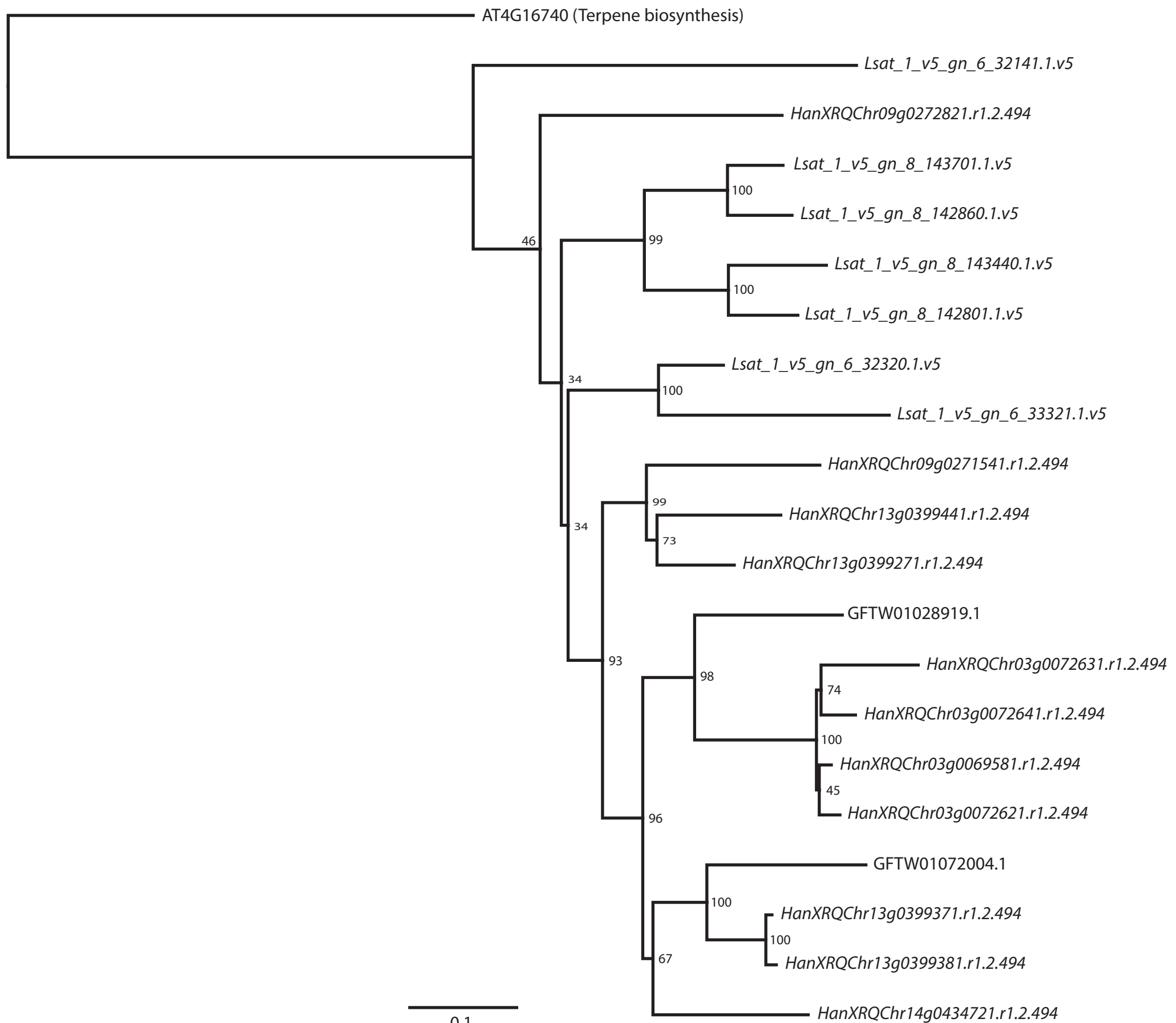

Additional file 7: Figure S4

Supplement: Supplementary file 7 — Additional file 7: Figure S4. Gene tree of the guayule gene GFTW010289191.1, a putative ortholog of Arabidopsis AT4G16740, a gene involved in terpene biosynthesis. [file 12870_2019_2106_MOESM7_ESM.pdf]
