# Supplementary material for: Transcriptomic and evolutionary analysis of the mechanisms by which P. argentatum, a rubber producing perennial, responds to drought
Source: BMC Plant Biol. 2019 Nov 13;19:494. doi: 10.1186/s12870-019-2106-2 (PMC6854645; doi:10.1186/s12870-019-2106-2)
Supplement: Supplementary file 2 — Additional file 2: Table S1. Mapping rate comparison between filtered and unfiltered guayule transcriptome. [file 12870_2019_2106_MOESM2_ESM.pdf]

Sample ID	Reads mapped to OT	Reads mapped to FT	Raw reads	% Mapped to OT	% Mapped to FT
S6_I100	9735457	9773527	11474575	84.84	85.18
S7_I100	12415299	12470125	16054937	77.33	77.67
S8_I100	12554161	12611211	15676860	80.08	80.44
S3_I25	15442603	15525613	18279264	84.48	84.94
S4_I25	11853799	11926947	16032615	73.94	74.39
S5_I25	9774707	9836335	14325788	68.23	68.66
Total	71776027	72143758	91844039	78.15	78.55

Supplemental Table 1. Read mapping rates against the original transcriptome (OT) from Stonebloom and Scheller vs the filtered transcriptome (FT). I100 and I25 refer to the irrigation scheme described in the text.
