# Supplementary material for: Transcriptomic and evolutionary analysis of the mechanisms by which P. argentatum, a rubber producing perennial, responds to drought
Source: BMC Plant Biol. 2019 Nov 13;19:494. doi: 10.1186/s12870-019-2106-2 (PMC6854645; doi:10.1186/s12870-019-2106-2)
Supplement: Supplementary file 4 — Additional file 4: Figure S2. qRT-PCR validation of the most differentially expressed guayule drought responsive genes. [file 12870_2019_2106_MOESM4_ESM.pdf]

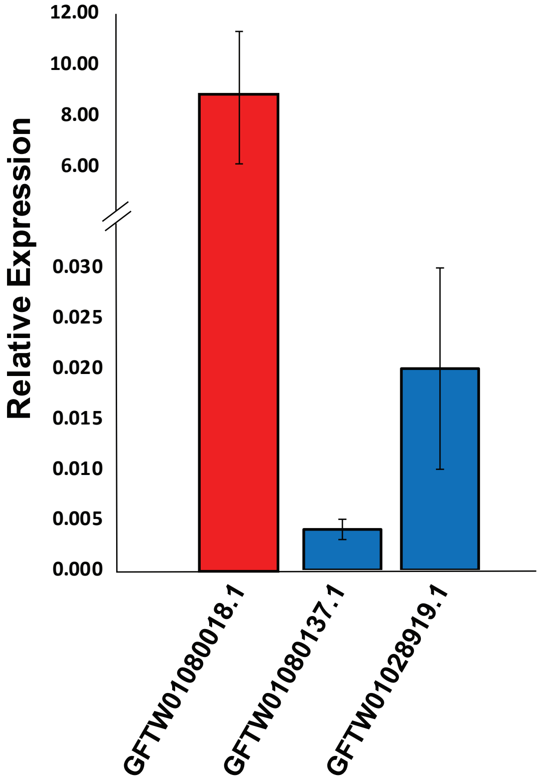

Additional file 4: Figure S2. qRT-PCR expression analysis of the most significantly up-regulated transcript GFTW01080018.1, the most significantly down-regulated transcript GFTW01080137.1, and the GFTW01028919.1 transcript which displayed the greatest decrease in transcription. Expression levels were calculated with the Livak and Schmittgen 2001 method, normalized to the 18S endogenous gene levels and compared to the calibrator (well-watered plant). Values are the average of three biological replicates, with corresponding standard deviation error bars.
